# A Differential Role for Macropinocytosis in Mediating Entry of the Two Forms of Vaccinia Virus into Dendritic Cells

**DOI:** 10.1371/journal.ppat.1000866

**Published:** 2010-04-22

**Authors:** Kerrie J. Sandgren, John Wilkinson, Monica Miranda-Saksena, Gerald M. McInerney, Karen Byth-Wilson, Phillip J. Robinson, Anthony L. Cunningham

**Affiliations:** 1 Centre for Virus Research, Westmead Millennium Institute, Sydney, New South Wales, Australia; 2 Faculty of Medicine, University of New South Wales, Sydney, New South Wales, Australia; 3 Department of Microbiology, Tumor and Cell Biology, Karolinska Institutet, Stockholm, Sweden; 4 Children's Medical Research Institute, Westmead, Sydney, New South Wales, Australia; University of Florida, United States of America

## Abstract

Vaccinia virus (VACV) is being developed as a recombinant viral vaccine vector for several key pathogens. Dendritic cells (DCs) are specialised antigen presenting cells that are crucial for the initiation of primary immune responses; however, the mechanisms of uptake of VACV by these cells are unclear. Therefore we examined the binding and entry of both the intracellular mature virus (MV) and extracellular enveloped virus (EV) forms of VACV into vesicular compartments of monocyte-derived DCs. Using a panel of inhibitors, flow cytometry and confocal microscopy we have shown that neither MV nor EV binds to the highly expressed C-type lectin receptors on DCs that are responsible for capturing many other viruses. We also found that both forms of VACV enter DCs via a clathrin-, caveolin-, flotillin- and dynamin-independent pathway that is dependent on actin, intracellular calcium and host-cell cholesterol. Both MV and EV entry were inhibited by the macropinocytosis inhibitors rottlerin and dimethyl amiloride and depended on phosphotidylinositol-3-kinase (PI(3)K), and both colocalised with dextran but not transferrin. VACV was not delivered to the classical endolysosomal pathway, failing to colocalise with EEA1 or Lamp2. Finally, expression of early viral genes was not affected by bafilomycin A, indicating that the virus does not depend on low pH to deliver cores to the cytoplasm. From these collective results we conclude that VACV enters DCs via macropinocytosis. However, MV was consistently less sensitive to inhibition and is likely to utilise at least one other entry pathway. Definition and future manipulation of these pathways may assist in enhancing the activity of recombinant vaccinia vectors through effects on antigen presentation.

## Author Summary

Vaccinia virus (VACV) is a relative of the smallpox virus and was used for many decades as a successful vaccine that contributed to the eradication of smallpox. Today, through genetic recombination technology, VACV shows potential as a modern vaccine for many unconquered diseases including HIV and cancer. Dendritic cells (DCs) are a specialised subset of immune cells that initiate adaptive immune responses and exploiting the interaction between VACV and DCs, which has not been well studied, may be a key to improving the efficacy of these vaccines. In this study we investigated the mechanisms by which VACV binds to and enters DCs. Here, we examined both the abundant mature virus form of VACV as well as the less common, poorly studied extracellular form. We found that VACV does not bind to the common pathogen-uptake C-type lectin receptors expressed on DCs and that the virus enters DCs via macropinocytosis—a fluid-phase uptake process. Furthermore, the virus is not delivered to the conventional endolysosomal antigen processing pathway in these cells. Our study provides new insights into VACV biology and into possible mechanisms of action of VACV as a recombinant viral vaccine vector which may assist in their rational design in the future.

## Introduction

Vaccinia virus (VACV) is best known for its role as a vaccine in the global eradication of smallpox. Research on VACV has been pursued with renewed fervour in recent years in light of its potential use as an effective vaccine vector for viral and parasitic infections as well as cancer. Exploiting certain aspects of the biology of the immune system may be the key to improving the efficacy of such modern vaccines.

Dendritic cells (DCs) are key players in the initiation of adaptive immune responses and as such are attractive targets for vaccination [1], [2]. They are specialised at antigen uptake and highly express C-type lectin receptors (CLRs), a family of Ca^2+^-dependent carbohydrate recognition receptors that bind to an array of microbial pathogens [3]. DCs use CLRs as a trapping mechanism for pathogens before internalisation or transfer of the pathogen to its specific receptor. DCs also employ a range of mechanisms for antigen uptake including receptor-mediated endocytosis and phagocytosis, as well as non-receptor-mediated processes such as macropinocytosis [4], [5]. Further information about the mechanisms of DC binding and uptake of VACV could be employed to better target VACV-vectored vaccines to DCs, either directly or via uptake of bystander infected cells and also influence recombinant antigen processing to enhance immune responses.

VACV is a large, enveloped DNA poxvirus that exists in multiple infectious forms [6], [7]. The majority of progeny virions are mature viruses (MV) which are released from the cell upon lysis. A small proportion of MVs become further enveloped and are exocytosed from the cell as extracellular virus (EV). The EV envelope contains unique viral proteins not found in the MV envelope [8]. As a result, MV and EV have been shown to have different binding characteristics and infection efficiencies [9]. Despite being studied for several decades, entry receptors for VACV have yet to be conclusively identified. MV binds to glycosaminoglycans [10]–[12] and also to the extracellular matrix protein laminin [13], however these interactions are not required for infection [13]–[16]. Furthermore, there is evidence that the receptor for VACV may differ between cell types such as primary haematolymphoid cells and epithelial cell lines [9], [17].

VACV enters cells via several different mechanisms in a cell-type specific manner [18]. Both forms of the virus can enter cell lines via fusion with the plasma membrane [14], [16], mediated by a multi-protein fusion complex on the virus [19], [20]. A low pH dependent endosomal route of entry and macropinocytosis have also been described for MV entry in cell lines [21]–[23]. An endocytic route of entry for EV has been suggested [24], [25] but not yet confirmed. Furthermore, in DCs, the visualisation of MV in intracellular vesicles by electron microscopy has suggested an endocytic mode of uptake [26] although the nature of these vesicles and the actual mode of uptake have not been described.

Few previous studies investigating VACV entry have examined EV as it represents only a small percentage of progeny virions and the outer membrane is very fragile [24] making purification and concentration of this form of the virus difficult. Here we have characterised the entry pathway for both MV and EV in human monocyte-derived dendritic cells (MDDCs) as a model for skin DCs. Using a systematic combination of pharmacological inhibitors and confocal microscopy we have shown that both forms of the virus are taken up via a clathrin-, caveolin-, flotillin- and dynamin-independent endocytic pathway, and the virus does not enter the endolysosomal pathway or rely on low pH to enter the cytoplasm. For EV, this uptake mechanism is predominantly macropinocytosis. MV is also macropinocytosed although to a lesser extent and it is likely that this form of VACV utilises multiple redundant entry mechanisms. In addition we have shown that VACV does not bind to CLRs expressed on DCs.

## Results

### Preparation of MV and EV stocks

To individually study the entry properties of MV and EV we first produced a concentrated, purified stock of GFP-labelled MV via ultracentrifugation on an Optiprep gradient. The purity of the stock was confirmed by immunofluorescence and electron microscopy, and SDS-PAGE followed by general protein staining and western blotting for the D8 and GFP proteins (Fig. S1). Although the fragility of the outer EV envelope makes purification of this virus difficult we were able to use gentle centrifugal filtration to produce a concentrated stock of GFP-labelled EV in which contaminating MV or damaged EV particles were subsequently neutralised with an MV-neutralising antibody (Fig. S2). The presence of intact EV in these preparations was confirmed by plaque assay in the presence of the neutralising antibody and also by immunofluorescence microscopy where intact EV was identified by direct detection with an EV-specific antibody or GFP-fluorescence as well as exclusion of MV-specific antibody staining (Fig. S3). On average, the percentage of intact EV was 46.0±15.9% with an intact EV titre of 2.3×10^7^ pfu/mL (n = 7). We used fresh EV preparations for each experiment and as the EV titre was calculated retrospectively, this resulted in a range of MOIs being used for EV experiments with multiple DC donors.

### VACV entry in MDDCs is dependent on factors consistent with an endocytic uptake mechanism

**Figure 1:**
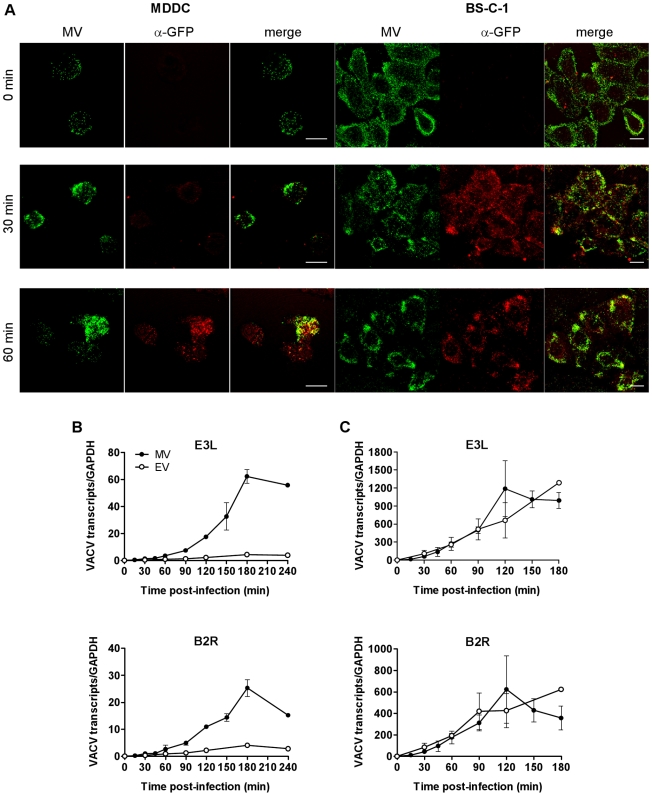
The release of VACV cores and initiation of viral gene expression is delayed in DCs compared to BS-C-1 cells. (A) MDDCs or BS-C-1 cells were spinoculated with MV-GFP (MOI 10) at 4°C then incubated at 37°C for 0, 30 or 60 min to allow virus entry. Cells were washed, fixed in 4% PFA and permeabilised with 0.1% Triton X-100 for 10 min at room temperature. MV was detected by confocal microscopy by GFP fluorescence (green) and virus cores were detected using an anti-GFP Ab and GAR-633 (red). Scale bars represent 10 µm. (B) MDDCs or (C) BS-C-1 cells were spinoculated with MV-GFP (MOI 3) or EV-GFP (MOI 1) at 4°C, washed and then incubated at 37°C for up to 4 h to allow virus entry. At various time points, cells were lysed and the expression of immediate early viral transcripts for two genes, E3L and B2R, was analysed by qPCR. Viral gene expression was normalised to GAPDH.

When we studied the kinetics of VACV entry in MDDCs we made a number of observations. Firstly, we observed virus capping at one end of the cells, a hallmark of endocytosis, for both MV (70% of 327 cells) and EV (67% of 90 cells) within 30 min of binding, consistent with a previous report [26]. We also observed that almost all MV bound to cells at 4°C could be stripped by trypsin, whereas 30 min after entry at 37°C, around half of the bound virions became resistant to trypsin. Moreover, virus cores could first be detected in the cytoplasm only after 60 min by probing with an anti-GFP antibody. This was in marked contrast to BS-C-1 cells where cores were readily detectable at 30 min (Fig. 1A). We interpret these results to suggest VACV does not fuse with the plasma membrane but is removed from the surface of the cell within 30 min and takes up to 60 min to fuse out of an intracellular compartment. VACV infection is abortive in DCs, limited to the expression of early viral proteins which takes place in the cytoplasm [26] but the delayed appearance of virus cores in DCs compared to BS-C-1 cells was mirrored in the kinetics of expression of two immediate early viral genes. E3L (a dsRNA-binding protein/PKR inhibitor) and B2R (unknown protein) transcripts were abundant within 15 min of virus entry, peaking at 2 h in BS-C-1 cells but were not clearly detectable in DCs until 45-60 min with a delayed peak at 3 h (Fig. 1B, C). Furthermore, the magnitude of early viral gene expression from MV and EV was equal in BS-C-1 cells but in DCs, gene expression from EV was suppressed compared to MV, suggesting differences in the entry pathways or the mechanics of virus core release between the cell types and possibly even between MV and EV in DCs.

**Figure 2:**
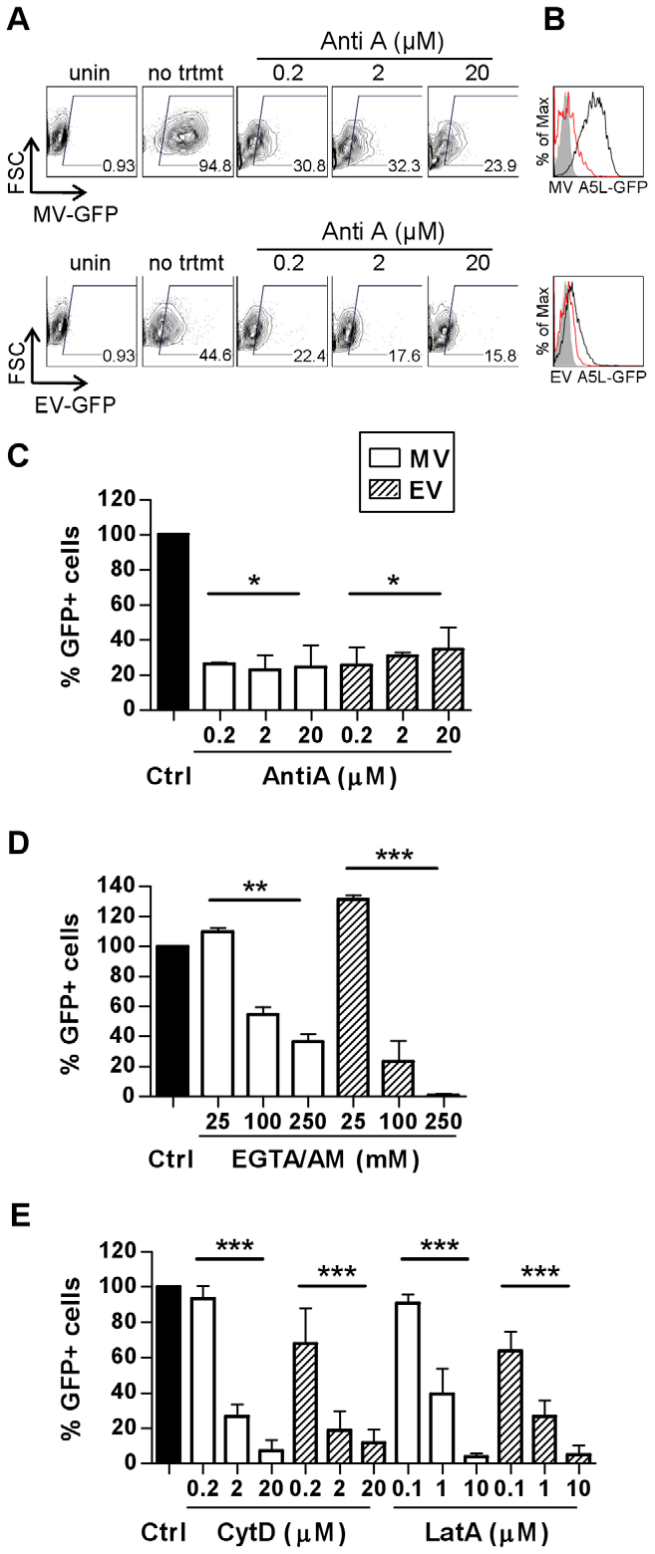
VACV entry in MDDCs is dependent on cellular factors consistent with an endocytic uptake mechanism. The effect of depletion of ATP (A–C) or intracellular calcium (D) and sequestration of actin (E) on the entry of MV (white bars) and EV (shaded bars) was assayed by FACS. MDDCs were pre-treated with inhibitors antimycin A (AntiA), EGTA/AM, cytochalasin D (CytD) and latrunculin A (Lat A) at the concentrations indicated for 60 min at 37°C prior to spinoculation with MV-GFP (MOI 20) or EV-GFP (MOI 0.5–5) at 4°C. Cells were shifted to 37°C for 30 min to allow virus entry then washed and the remaining surface-bound virus stripped by trypsinisation. The percentage of GFP-positive cells was analysed by flow cytometry and representative data is shown for AntiA in (A) dot plot and (B) histogram form with uninfected (shaded), untreated, infected control (black line) and 20 µM AntiA treatment (red line) overlaid. The EV MOI in this experiment was 1.2. (C–E) Data from three independent experiments was normalised as a percentage of the untreated control. * p<0.05. ** p<0.01. *** p<0.001.

Active uptake of antigen by DCs is an energy-intensive process requiring rearrangement of the plasma membrane and cytoskeleton and ligation of cellular receptors which often triggers a signalling cascade that coordinates internalisation of the antigen by endocytosis and subsequent events. Conversely, fusion of a viral envelope with the plasma membrane does not usually require cellular ATP and may or may not induce signalling. To distinguish between these pathways of entry in DCs, we examined the requirement for ATP for VACV entry using antimycin A (AntiA), an inhibitor of the mitochondrial electron transport chain that has been shown to inhibit energy-dependent processes [27]. MDDCs were pre-treated with AntiA in glucose-free medium, prior to spinoculation with GFP-labelled MV or EV at 4°C. Bound virus was then allowed to enter the cells in the presence of inhibitor for 30 min at 37°C and any remaining surface-bound virus was stripped by trypsinisation. We chose a 30 min time point to specifically assess the drug's effect on the initial step of virus entry into vesicles. Virus entry was measured by detection of GFP by flow cytometry. Concentrations up to 20 µM AntiA depleted cellular ATP in a dose-dependent fashion by up to 95% (data not shown). Both MV and EV entry was significantly reduced in AntiA-treated cells compared to untreated cells, up to 77.2±8.7% (mean ± SEM, n = 3, p = 0.029) for MV and 74.6±10.4% (n = 3, p = 0.029) for EV (Fig. 2A–C), although the remaining ∼25% remained refractory to increasing concentrations of AntiA. Thus, VACV depends on cellular energy for entry in MDDCs.

Next, as ligation of cellular receptors often triggers a Ca^2+^-mediated signalling cascade that coordinates internalisation of the antigen and subsequent events, we examined VACV entry into MDDCs in the presence of EGTA/AM, a membrane permeable intracellular Ca^2+^ chelator. Both MV and EV entry was dependent on Ca^2+^ (Fig. 2D). MV entry was significantly inhibited by 63.4±4.7% (n = 4, p = 0.003) in the presence of 250 µM EGTA/AM whereas EV entry was almost completely abrogated (99.1±0.7%, n = 3, p<0.001). Interestingly, low concentrations of EGTA/AM (2.5-25 µM) consistently enhanced both MV and EV entry by 10– 0%. Treatment of the cells with non-membrane permeable EGTA had no inhibitory effect on VACV entry (data not shown) indicating that it was intracellular Ca^2+^ stores that were important for virus entry.

Many viruses rely on dynamic changes to the actin cytoskeleton to aid their entry, either to effect endocytosis [28] or transport membrane-bound virus to areas of high endocytic activity [29]. The drugs cytochalasin D (CytD) and latrunculin A (LatA) disrupt actin polymerisation and inhibit these processes [30]. Both MV and EV entry into MDDCs was significantly inhibited, in a dose-dependent manner, by more than 88.4% in treated cells compared to untreated cells at the highest concentrations of both CytD and LatA (n = 3, p<0.001 for all; Fig. 2E). These data indicate that there is a requirement for actin cytoskeleton rearrangements in VACV entry into MDDCs.

Altogether, the delayed appearance of virus cores, a reliance on cellular ATP, intracellular Ca2^+^ and actin strongly suggests that VACV is taken up actively, via an endocytic or macropinocytic mechanism in MDDCs.

### VACV does not utilise C-type lectin receptors (CLRs) for binding to MDDCs

**Figure 3:**
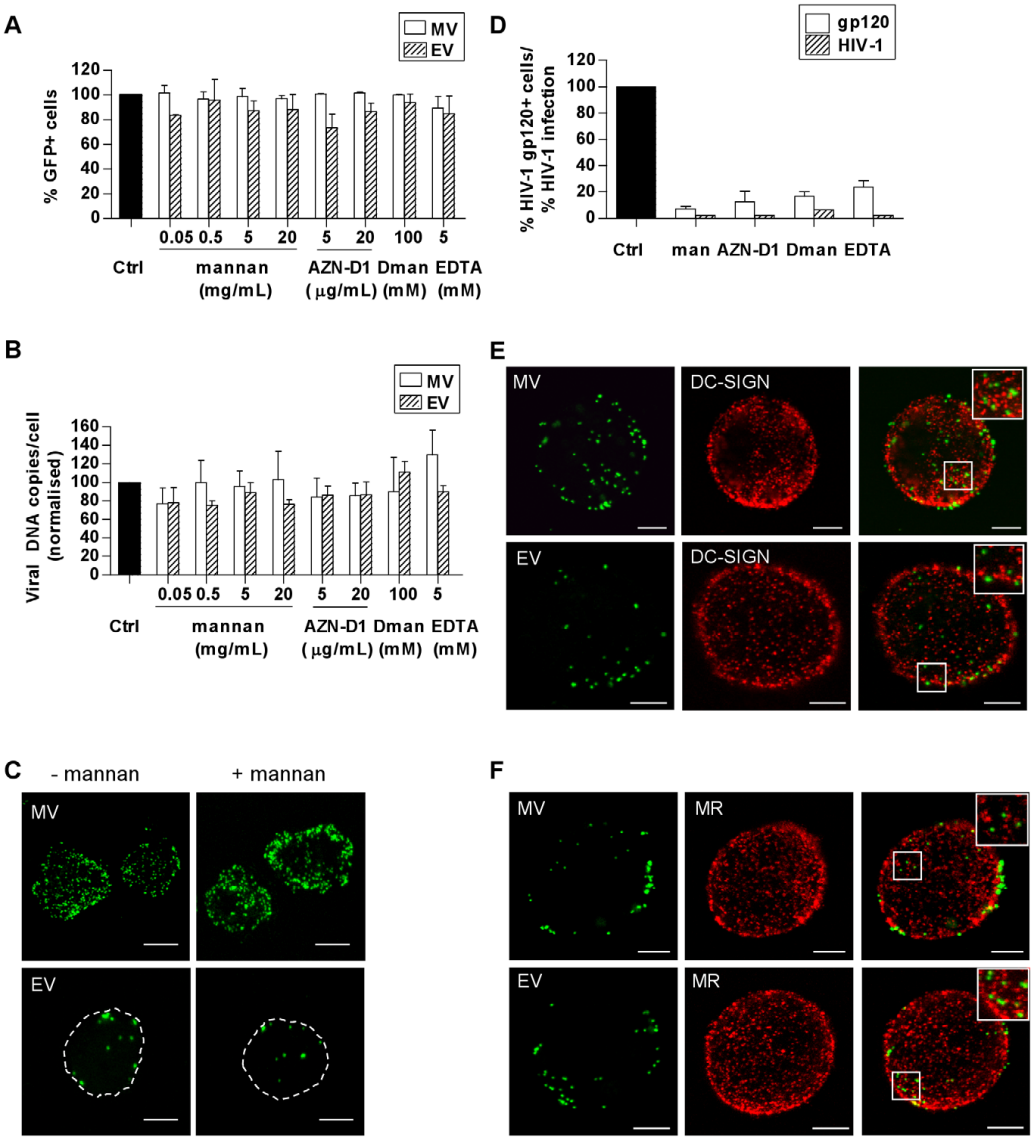
VACV does not bind to C-type lectin receptors (CLRs) expressed on MDDCs. MDDCs were pre-treated with the CLR inhibitors mannan, a neutralising anti-DC-SIGN mAb (AZN-D1), D-mannose (D-man) and EGTA or media alone at 4°C for 30 min then spinoculated at 4°C with MV-GFP (MOI 50) or EV-GFP (MOI 5–10). Cells were then washed and fixed in 4% PFA for flow cytometry or lysed for qPCR. (A) The percentage of GFP-positive cells was analysed by flow cytometry and normalised as a percentage of the untreated control. (B) Virally-encoded GFP DNA copy numbers were quantitated by qPCR and normalised to GAPDH and expressed as a percentage of the untreated control. (C) Confocal microscopy images of MV or EV bound to MDDCs at 4°C in the absence or presence of mannan. (D) As a positive control, pre-treated MDDCs were incubated with biotinylated HIV-1 gp120 at 4°C which was detected with streptavidin-PE and measured by flow cytometry (white bars). Alternatively, cells were infected with HIV-1 (MOI 10) in the presence of inhibitors for 72 h then integrated viral transcripts were measured by qPCR (shaded bars). (E, F) Colocalisation of MV or EV on the surface of MDDCs with DC-SIGN and MR was assessed by confocal microscopy. DC-SIGN and MR mAbs were detected with GAM-546. Images are maximum projections of z-series and the scale bars represent 5 µm. Inserts are enlargements of the boxed areas in the main images.

DCs express an array of CLRs that mediate rapid endocytosis of a variety of glycosylated antigens and pathogens in a Ca^2+^-dependent manner [3]. Mannose receptor (MR) and DC-SIGN are two CLRs that are highly expressed on MDDCs. After having established that VACV is likely taken up via some form of endocytosis in MDDCs, we investigated whether these CLRs were involved in this process. MDDCs were treated with a variety of CLR inhibitors–mannan (a pan-CLR inhibitor), a neutralising anti-DC-SIGN mAb, D-mannose (a specific inhibitor for MR) and EGTA (a Ca^2+^ chelator), prior to MV or EV binding at 4°C. Virus binding was measured by flow cytometry of GFP fluorescence (Fig. 3A) or qPCR detection of the virally encoded GFP gene (Fig. 3B), or qualitatively by confocal microscopy (Fig. 3C). Binding of either MV or EV was not significantly reduced by any of the CLR inhibitors nor was there any evidence of a dose-response to mannan. In contrast, the inhibitors were effective at blocking HIV-1 gp120 binding to MDDCs as well as HIV-1 infection of MDDCs (Fig. 3D), as previously reported [31]. Furthermore, VACV bound to the surface of MDDCs did not colocalise with DC-SIGN or MR (Fig. 3E, F). There was also no appreciable difference in the number of particles bound to DC-SIGN- and MR-bright cells compared to dim cells. Thus we conclude that mannose-binding CLRs are not involved in the binding and entry of VACV to MDDCs.

### VACV enters MDDCs via a clathrin- and caveolin-independent mechanism

Next we investigated whether clathrin-mediated or caveolin-mediated endocytosis are involved in VACV uptake in DCs. VACV has dimensions of 250–350 nm, which possibly precludes it from clathrin- or caveolin-mediated endocytosis as these pathways are normally reserved for particles with a diameter of 100 nm or less. However, these size restrictions may not be absolute particularly in DCs that are specialised at antigen uptake.

**Figure 4:**
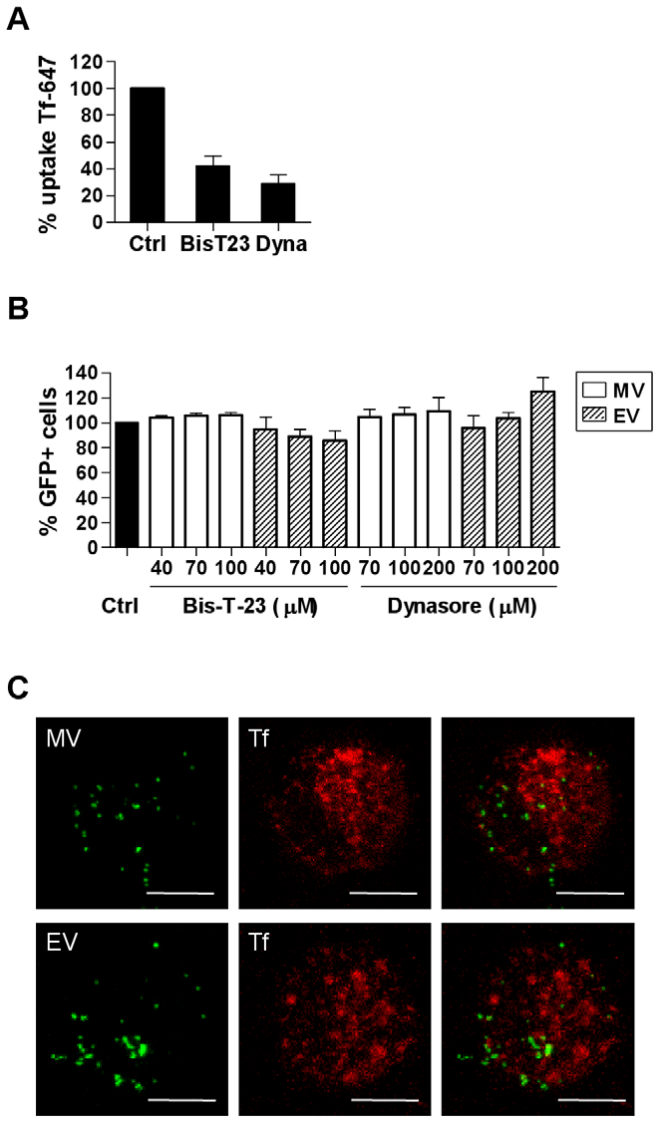
VACV entry in MDDCs does not require dynamin. (A) MDDCs were pretreated with dynamin II inhibitors Bis-T-23 (100 µM) and Dynasore (Dyngo7a; 200 µM) or media alone at 37°C for 30 min then incubated with 5 µg/mL transferrin-Alexafluor 647 (Tf-647) for 10 min at 37°C. Cells were washed, and then any remaining surface-bound transferrin was stripped by a low pH wash (pH 2.8). Uptake of transferrin was measured by flow cytometry and the mean fluorescence intensity (MFI) was expressed as a percentage of the untreated control MFI. (B) Alternatively, MDDCs were pre-treated with the dynamin II inhibitors then infected with MV-GFP or EV-GFP and analysed, as in Fig. 2. (C) MDDCs were spinoculated with MV-GFP or EV-GFP at 4°C then incubated in pre-warmed media with 200 µg/mL Tf-647 at 37°C for 1-45 min. Cells were then washed and trypsinised to remove residual surface bound virus and fixed in 4% PFA for confocal microscopy. Representative maximum projections of z-series taken at 15 min are shown. Scale bars represent 5 µm.

The large GTPase dynamin II is required for pinching off endocytic vesicles from the plasma membrane during clathrin-mediated and caveolin-mediated endocytosis [32], [33]. We used two dynamin inhibitors–Bis-T-23 and Dynasore (Dyngo7a) that act via different mechanisms to study the requirement for dynamin in VACV entry. Dynasore is a dynamin GTPase inhibitor while Bis-T-23 acts on the assembly domain of dynamin. Each of these drugs inhibited uptake of transferrin, which is taken up by clathrin-mediated endocytosis via the transferrin receptor (Fig. 4A), but did not inhibit MV uptake (Fig. 4B). EV uptake was slightly but not significantly inhibited in the presence of Bis-T-23 (14.3±7.7%, n = 3, p = 0.075) and no inhibitory effect was seen with Dynasore (Fig. 4B). Furthermore, when VACV was bound to MDDCs at 4°C then allowed to enter in the presence of fluorescently conjugated transferrin, no colocalisation occurred between the two antigens over a course of 30 min at 37°C (Fig. 4C). Thus, VACV entry in DCs is dynamin-independent.

We also found that while caveolin-1 (Cav-1) expression was detectable in MDDCs at the RNA level, only very low amounts of Cav-1 protein were detected by western blot which were undetectable by flow cytometry or confocal microscopy (data not shown). Therefore since VACV entry does not require dynamin, does not colocalise with transferrin and Cav-1 expression is almost undetectable in MDDCs, we conclude that VACV entry in DCs is not via clathrin- or caveolin-mediated endocytosis.

### VACV entry in MDDCs is cholesterol-dependent

**Figure 5:**
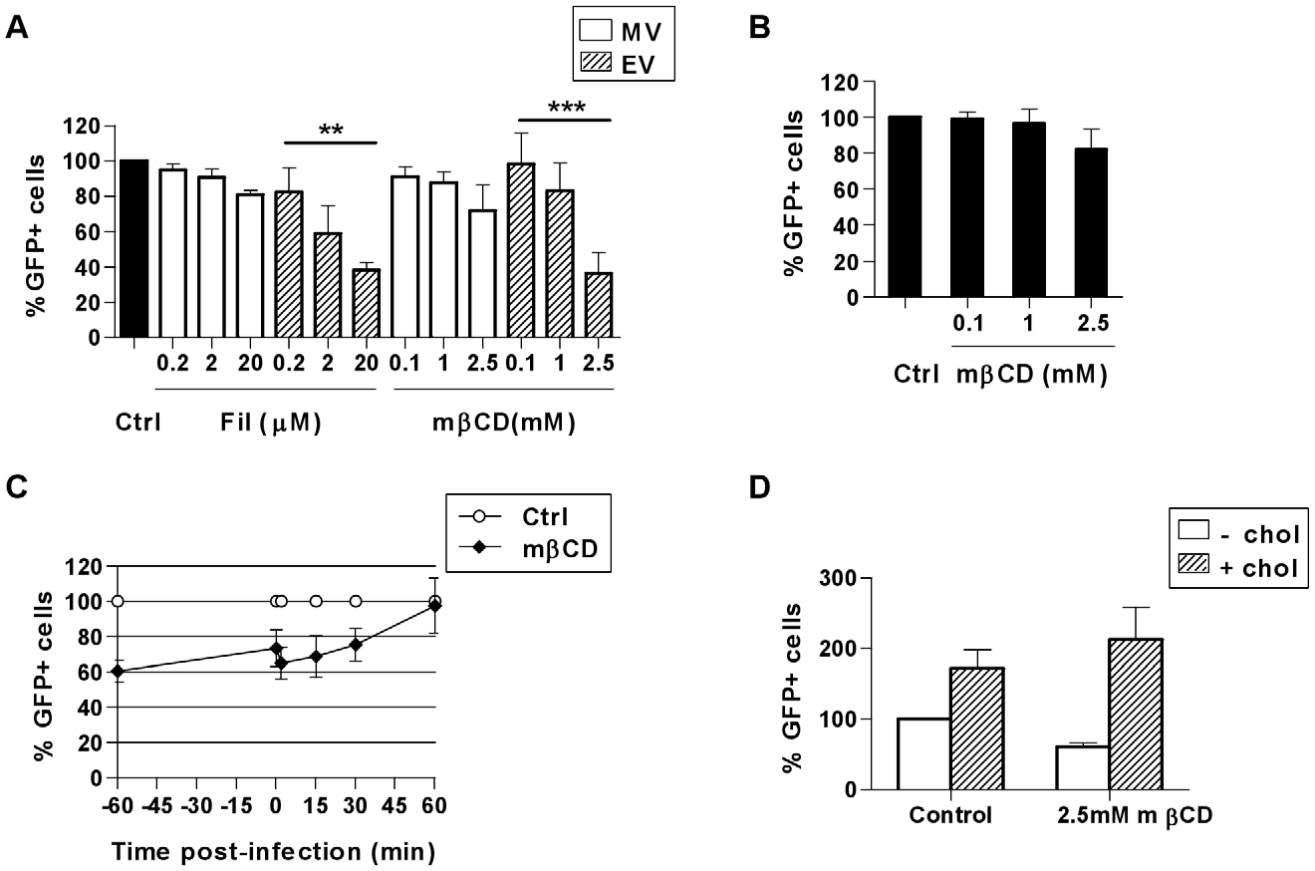
VACV entry in MDDCs is dependent on cholesterol. (A) Cells were treated with cholesterol inhibitors methyl-β-cyclodextrin (mβCD) or filipin III (Fil) for 60 min at 37°C then infected and analysed as in Fig. 2. (B) Alternatively, virus binding at 4°C was analysed immediately after spinoculation of mβCD-treated cells with MV-GFP or (C) mβCD was added at the times indicated from 60 min prior to, up to 60 min post MV binding and entry. (D) Following treatment with mβCD for 60 min, cells were washed and incubated for a further 60 min at 37°C in the absence or presence of 0.1 mM soluble cholesterol to replenish cellular cholesterol, followed by MV binding and entry as in (A). ** p<0.01. *** p<0.001.

An alternative endocytic pathway is the clathrin-independent carrier (CLIC) pathway which is clathrin-, caveolin- and dynamin-independent but requires cholesterol in the plasma membrane [4], [5]. Macropinocytosis also occurs in cholesterol-rich domains in the cell membrane [34]. We used two agents to disrupt lipid rafts to determine their involvement in VACV entry in MDDCs. Methyl-β-cyclodextrin (mβCD) disrupts lipid rafts by extracting cholesterol from lipid membranes and filipin III (Fil) is a sterol-binding agent that sequesters cholesterol within the membrane. We observed notable reductions in MV entry of up to 19.1±2.2% (n = 3) in the presence of Fil and up to 28.3±14.6% (n = 3) in the presence of mβCD, however these reductions were not statistically significant (p = 0.183 and 0.305 respectively. Fig. 5A). Conversely, EV entry was significantly reduced in the presence of each drug in a dose-dependent manner by 61.9±4.3% (n = 4, p<0.001) and 63.4±11.6% (n = 4, p = 0.009) compared to untreated cells (Fig. 5A). The previously reported finding that MV penetration of BSC40 cells can be reduced by more than 90% using 10 mM mβCD [35] could not be repeated in MDDCs since concentrations greater than 2.5 mM were found to be toxic to these cells (data not shown).

The concentrations of mβCD used here reduced the content of cellular cholesterol by up to 30% compared to the untreated control, as measured by an Amplex Red assay (data not shown), however the cholesterol content could not be further reduced due to the toxicity of higher drug concentrations. While MV was clearly less sensitive to cholesterol-depletion than EV, we wanted to confirm our hypothesis that MV entry might be increasingly inhibited if the MDDCs could tolerate more marked cholesterol depletion. To further elucidate the requirement for cholesterol in MV entry in MDDCs we firstly pre-treated the cells with mβCD, and found MV binding at 4°C was moderately inhibited by 17.9±11.1% (n = 5, Fig. 5B). Secondly, mβCD was added to cells either 60 min prior to, at the time of, or up to 60 min after the initiation of virus entry at 37°C. Once MV entry was initiated, the degree to which the addition of mβCD could inhibit entry was diminished until finally at 60 min post-entry, mβCD no longer had any effect on virus uptake (Fig. 5C). The partial recovery of MV entry that occurred between treatment at −60 min and 0 min may be indicative of redistribution of the remaining cholesterol in the plasma membrane that was sufficient for MV entry. Thirdly, in the presence of mβCD, we were able to rescue and indeed enhance the entry of MV in MDDCs with the addition of soluble cholesterol (Fig. 5D). Together these results show that cholesterol indeed contributes to MV entry in DCs.

### VACV entry is independent of flotillin-1 (Flot-1)

The CLIC pathway is the major pathway for the uptake of cholera toxin B and GPI-linked proteins and is marked by high concentrations of Flot-1 in the plasma membrane and the membranes of endocytic intermediates [4], [36]. We found that MDDCs express Flot-1 at the RNA and protein level (Fig. S4). However, when VACV was bound to MDDCs at 4°C then allowed to enter over a course of 60 min, neither MV nor EV colocalised with Flot-1 at the time of virus binding or during entry (Fig. S4). Thus the entry of VACV does not appear to be associated with Flot-1.

### VACV entry is independent of Fc and complement receptors

DCs employ phagocytosis for the ingestion of large (>500 nm) particulate antigens via Fcγ and complement receptors. We used aggregated IgG and a neutralising CD18 mAb respectively to block each of these receptors and found no reduction in the binding or entry of MV or EV in MDDCs (data not shown). However, phagocytosis via other receptors, such as scavenger receptors, cannot be ruled out at this stage.

### VACV can enter MDDCs via macropinocytosis

Macropinocytosis is a non-receptor mediated pathway characterised by the extension of filopodia that fold back onto the cell membrane to form large, irregular macropinosomes. It is a constitutive process in DCs enabling them to sample and concentrate large quantities of soluble antigen and contributes to efficient MHC class I and class II presentation to T cells [37], [38]. Macropinocytosis also provides a means of entry into host cells for several pathogens including bacteria [39], [40] and viruses [41]–[44]. As mentioned previously, the MV form of VACV enters HeLa cells via macropinocytosis [23], [45]. Macropinocytosis is dependent on ATP, actin and an increase in intracellular Ca^2+^ for membrane ruffling and the formation of filopodia and depends on cholesterol but not dynamin [34], [46], [47].

**Figure 6:**
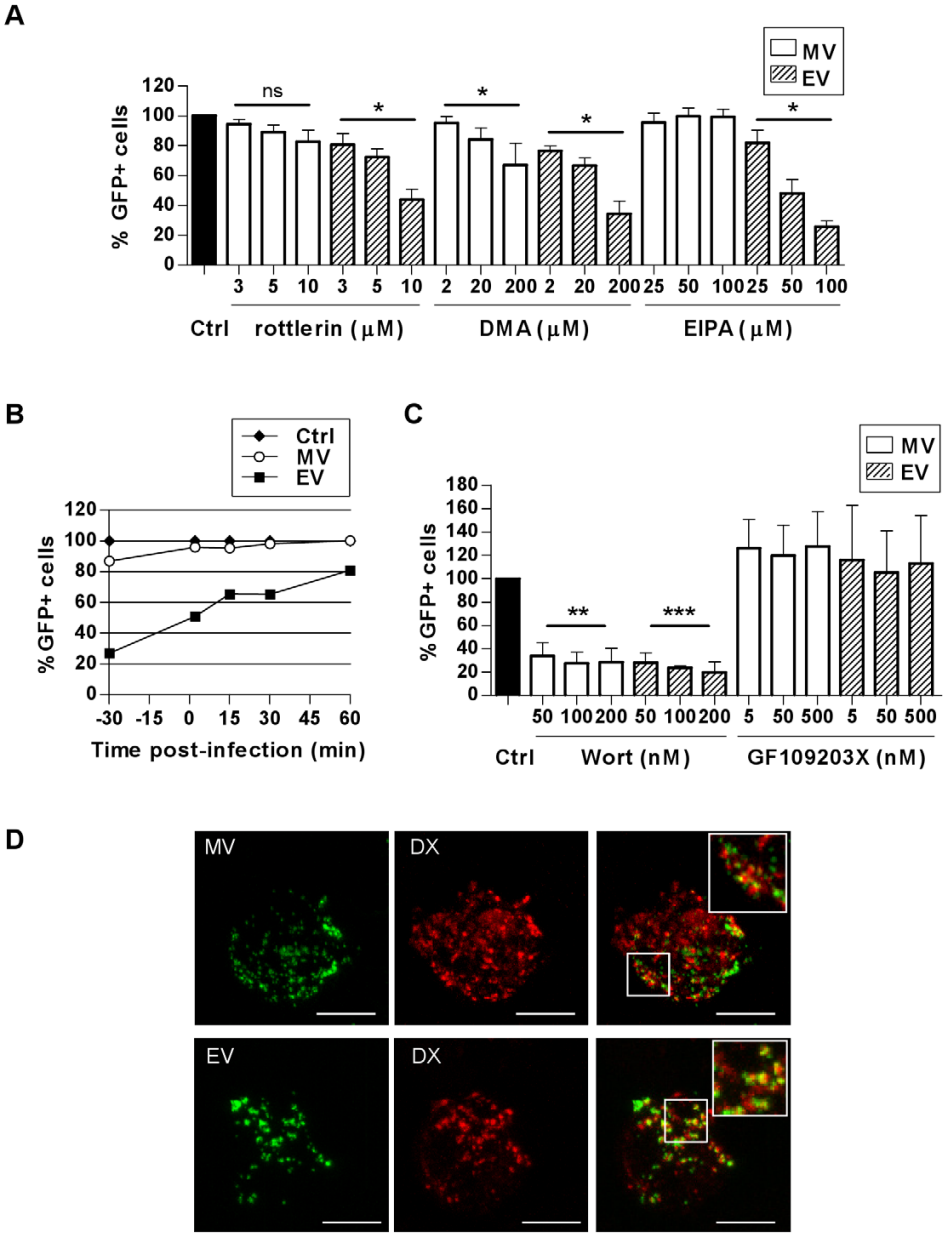
VACV is taken up by macropinocytosis in MDDCs. The effect of (A) macropinocytosis inhibitors rottlerin, DMA and EIPA or (C) kinase inhibitors wortmannin (wort) or GF109203X on the entry of MV-GFP and EV-GFP was assayed by FACS. MDDCs were pretreated with inhibitors at the concentrations indicated for 30 min at 37°C then infected and assayed as in Fig. 2. * p<0.05, ** p<0.01, *** p<0.001. (B) The kinetics of rottlerin inhibition were investigated. Cells were treated with 10 µM rottlerin at the times indicated relative to infection with MV-GFP or EV-GFP, then assayed as in Fig. 2. (D) MV and EV colocalise with dextran during uptake by MDDCs. Cells were spinoculated with MV-GFP or EV-GFP at 4°C then incubated in pre-warmed media with 0.5 mg/mL dextran-Texas Red at 37°C for up to 30 min. Cells were then washed and trypsinised to remove residual surface bound virus and fixed in 4% PFA for confocal microscopy. Representative maximum projections of z-series taken at 15 min are shown. Scale bars represent 5 µm. Inserts are enlargements of the boxed areas in the main images.

To test whether macropinocytosis is also involved in VACV entry in DCs we used three commonly used inhibitors of macropinocytosis–rottlerin, 5-(N-ethyl-N-isopropyl) amiloride (EIPA) and dimethyl amiloride (DMA). Amilorides inhibit the Na^+^/H^+^ ion exchange pump in the plasma membrane affecting the intracellular pH, resulting in the cessation of macropinocytosis, however the mechanism of rottlerin inhibition is unknown [48]. EV entry was significantly reduced by 56.3±6.9% (n = 3, p = 0.023) with rottlerin, 65.8±8.6% (n = 3, p = 0.019) with DMA and 74.3±3.9% (n = 3, p = 0.038) with EIPA. In contrast, MV entry was modestly reduced in the presence of rottlerin (17.4±7.8%, n = 3, p = 0.06) and DMA (33.9±14.9%, n = 3, p = 0.020) but remained unaffected by EIPA compared to untreated cells (Fig. 6A). We tested the specificity of the three drugs for macropinocytosis in MDDCs by assessing their ability to inhibit uptake of the classical fluid-phase marker Lucifer Yellow, without affecting transferrin uptake which is via receptor-mediated endocytosis (Fig. S5). Both rottlerin and DMA effectively inhibited Lucifer Yellow uptake, however DMA equally inhibited transferrin uptake. In contrast, rottlerin had only a minimal effect on transferrin uptake, consistent with previous reports [48]. EIPA was actually more effective at inhibiting transferrin than Lucifer Yellow uptake. Therefore in MDDCs, rottlerin can be considered a specific macropinocytosis inhibitor, whereas the other two drugs are less specific. Since others have reported that EIPA blocks uptake of MV in HeLa cells [23] we determined whether the effect of this drug was cell type-dependent. Despite having no effect on MV uptake in MDDCs, EIPA did indeed block uptake of MV and EV in HeLa cells (Fig. S5), consistent with the previous report, demonstrating that the effects of EIPA are cell type-dependent.

We wanted to further investigate whether the small reduction in MV entry as a result of rottlerin treatment was similar to that observed with the cholesterol inhibition studies where MV entry was less sensitive to perturbation than EV and the MDDCs were able to partially recover from the effects of the drug treatment sufficiently for MV entry to occur. To address this, we used a drug treatment time course, adding rottlerin either 30 min before, at the time of, or up to 60 min after initiating virus entry at 37°C. EV entry was clearly blocked with rottlerin treatment prior to virus entry and the effect diminished as rottlerin was added at later times after virus entry had begun to take place (Fig. 6B). Although the effect on MV was less significant, the trend was the same as for EV which suggests that a proportion of MV is entering via macropinocytosis. Additionally, we assessed whether using a higher MOI with MV was masking the effect that was visible with EV with these and several other of the entry inhibitors we have employed but when we repeated the experiments using a high MOI (10) versus a low MOI (1) for MV the results were identical (data not shown).

Macropinocytosis in various cell types is dependent on several kinases, including phosphotidylinositol-3-kinase (PI(3)K) and protein kinase C (PKC) which are involved in the signalling pathways that promote membrane ruffling and macropinosome formation, although it has been shown that PKC is not critical for macropinocytosis in DCs [48]. We found that both MV and EV entry in MDDCs was significantly reduced in the presence of wortmannin, a PI(3)K inhibitor, but not in the presence of GF109203X, a small molecule inhibitor of PKC (Fig. 6C). MV entry was decreased by up to 72.5±9.3% (n = 3, p = 0.003) and EV by 80.3±8.8% (n = 3, p<0.001) by wortmannin, implicating the involvement of PI(3)K in VACV entry in MDDCs.

Finally, we examined VACV uptake in the presence of another fluid-phase marker, high molecular weight dextran, by confocal microscopy. We observed that both MV and EV considerably colocalised with dextran-Texas Red (Fig. 6D). After 15 min this was 29.3±10.8% and 29.7±9.5% for MV and EV respectively, increasing to 42.5±3.0% and 35.1±3.3% respectively after 30 min. Having established that VACV entry in MDDCs is dependent on ATP, actin, intracellular Ca^2+^ and cholesterol but not dynamin (all features of macropinocytosis), the pattern of inhibition of VACV entry by rottlerin and DMA and reliance on PI(3)K together with its colocalisation with dextran strongly suggest that VACV enters DCs by macropinocytosis. For EV this is the major route of entry however, it appears that this is a sub-dominant pathway for MV and it is likely that this form of the virus utilises multiple, redundant entry mechanisms.

### VACV does not traffic through the endolysosomal route or rely on low pH to enter the cytoplasm

**Figure 7:**
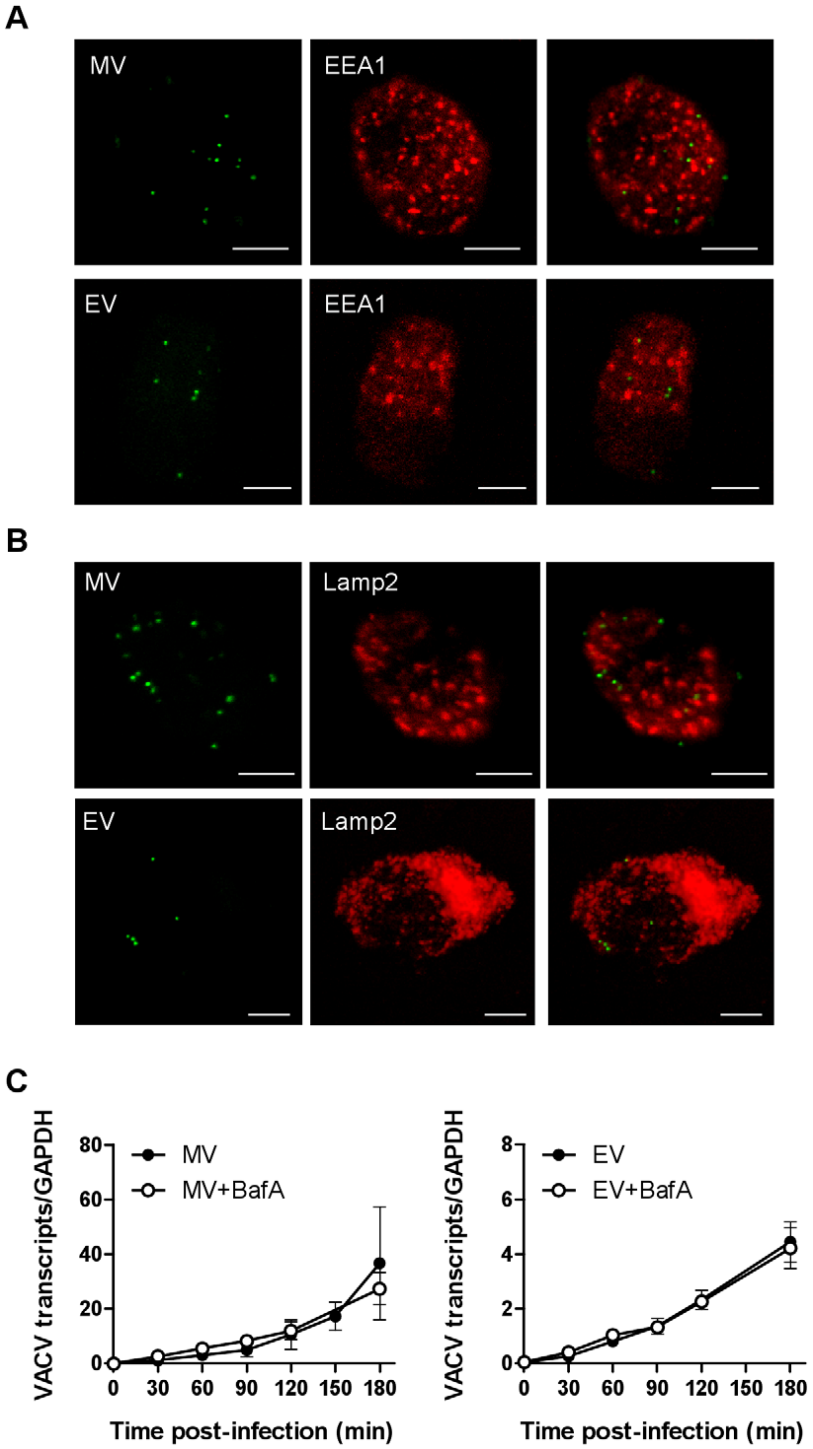
VACV does not colocalise with endolysosomal markers and is not dependent on low pH to enter the cytoplasm. MDDCs were spinoculated with MV-GFP (MOI 10) or EV-GFP (MOI 2–5) at 4°C. After washing to remove any unbound virions, cells were incubated at 37°C to allow virus entry for 0-120 min. Cells were then fixed and permeabilised with methanol:acetone (1∶1 v/v) for 2 min at -20°C and stained for (A) early endosomes using EEA1 mAb or (B) lysosomes with Lamp2 mAb, and GAM-546. Maximum projections of z-series for EEA1 at 30 min and Lamp2 at 60 min are shown and are representative of three different donors. Scale bars represent 5 µm. (C) MDDCs were spinoculated MV-GFP (MOI 3) or EV-GFP (MOI 1), washed and incubated at 37°C in the presence or absence of 250 nM bafilomycin A (BafA) for up to 3 h. At various time points, cells were lysed and the expression of the immediate early viral gene E3L was analysed by qPCR. Viral gene expression was normalised to GAPDH.

**Figure 8:**
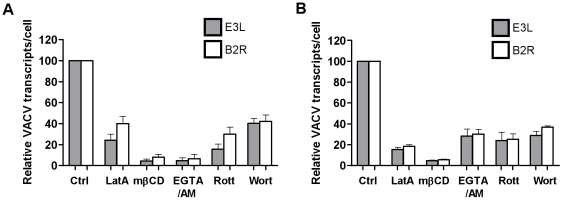
Blocking components of the macropinocytosis pathway blocks viral gene transcription. MDDCs were pre-treated with the LatA (10 µM), mβCD (2.5 mM), EGTA/AM (250 mM), rottlerin (10 µM) and wortmannin (200 nM) for 30 min prior to spinoculation with (A) MV-GFP (MOI 3) or (B) EV-GFP (MOI 1) at 4°C. Cells were washed and incubated at 37°C to allow virus entry for 2 h then lysed for analysis of immediate early viral transcripts E3L (shaded bars) and B2R (white bars) by qPCR. The expression of viral transcripts was normalised to expression of GAPDH and is expressed as a percentage of the untreated control from two independent donors with standard error bars.

Finally, we investigated the fate of VACV upon entry into DCs. Little is known about the trafficking of macropinosomes, however, their destination seems to depend on the nature of the cargo [49]. We looked for colocalisation of the virus with the early and late endosomal markers, EEA1 and CD63 and the lysosomal marker, Lamp2. Neither MV nor EV colocalised significantly with EEA1 over the course of 60 min of virus entry (Fig. 7A). Furthermore, neither form of VACV colocalised with CD63 (data not shown) or Lamp2 (Fig. 7B). We also assessed the effect of bafilomycin A which prevents the acidification of intracellular compartments, on viral gene transcription and found it to be unaltered for two genes for both MV and EV (Fig. 7C and data not shown). This was consistent with the fact that we did not see any degradation or quenching of the viral GFP signal by flow cytometry over time, in the presence or absence of bafilomycin A, that would be induced if the virus accessed an acidic compartment. Finally, to confirm that the macropinocytic pathway we had been inhibiting in our flow cytometry assay does in fact lead to bona-fide infection of the DCs, we measured viral gene expression in the presence of representative inhibitors. LatA, mβCD, EGTA/AM, rottlerin and wortmannin all blocked immediate early gene transcription for both MV and EV (Fig. 8). Thus VACV is taken up by macropinocytosis into a compartment that is distinct from the endolysosomal pathway in DCs and does not depend on low pH to release virus cores into the cytoplasm where viral gene transcription takes place.

## Discussion

Elucidating the mode of uptake of VACV by DCs is necessary in order to fully understand the biology of the virus and also vaccine systems that involve VACV vectors. VACV infection of DCs induces apoptosis, but this is somewhat delayed, not occurring until 48 h after infection with WR strain. Infection in DCs is abortive and limited to the production of early proteins but direct presentation of viral antigens by infected DCs can be detected in the lymph nodes between 6–24 h hours following infection [50], [51]. It is highly likely that epidermal and perhaps dermal DCs are infected *in vivo*, in addition to keratinocytes, and contribute to direct priming of CD8^+^ CTLs. Expression of early viral genes may also trigger cytoplasmic pattern-recognition receptors in DCs and influence subsequent antigen presentation to bystanders. Furthermore, in the case of virus entry that does not result in “productive” infection (in the sense of viral gene transcription), the compartment the virus enters in DCs will also determine whether MHC class II loading and presentation occurs. Thus the route of VACV entry into these cells is of critical importance to understanding the immunobiology of VACV.

With respect to future VACV-based vaccines, MV is the likely important form of the virus. However, a replication-competent vaccine would lead to the production of EV in target cells at the site of vaccination and as these particles are thought to be responsible for long-range spread of the virus within the body and are more resistant to antibody neutralisation, DC capture and presentation of this form of VACV to T cells may play an important role in containing and limiting the infection. Thus both virus forms need to be studied.

The present study builds on the previous report of Drillien *et al* [26], who observed MV inside vesicles in MDDCs, to further define the entry pathway of VACV in these specialised antigen presenting cells. We have shown that VACV is taken up by MDDCs via an endocytic pathway that is independent of clathrin, caveolin, dynamin and flotillin but is dependent on ATP, actin, intracellular calcium, host cell membrane cholesterol and PI(3)K. The pathway is not mediated by CLRs and does not deliver VACV to early endosomes or lysosomes or progress to an acidic pH. For EV, this pathway is predominantly macropinocytosis. However, MV was consistently more resistant to entry inhibitors and whilst macropinocytosis contributed to a proportion of MV entry, this form of VACV likely uses multiple entry pathways in MDDCs including other clathrin- and caveolin-independent endocytic pathways. Our knowledge of the intricacy of endocytic pathways utilised by mammalian cells is rapidly expanding. Along with the well-defined clathrin- and caveolin-dependent pathways characterised by their requirement for dynamin, additional dynamin-independent pathways, separated by their dependence on various small GTPases (Rac1, Cdc42, ARF6) are beginning to be defined [4], [5]. Macropinocytosis and the CLIC pathway fall into the latter category. Phagocytosis is generally discriminated from other forms of endocytosis by the size of the particle being ingested and by morphological features–the extension of pseudopods around the particle rather than the invagination of the cell membrane, and the close-fitting nature of the phagosome due to multiple receptor-particle interactions. With dimensions of 250–350 nm, VACV falls between the generally accepted upper size limit of endocytosis (100 nm) and just below the lower size limit of phagocytosis (500 nm). Macropinosomes however can range in size from a few hundred nanometers up to several micrometers in diameter [38].

In addition to the size restrictions on clathrin- and caveolin-mediated endocytosis, our data has ruled out these modes of uptake for VACV entry in MDDCs. The cholesterol inhibitor Fil, which does not affect clathrin-mediated endocytosis [52], did have an effect on the entry of both MV and EV and we found expression of caveolin-1 to be undetectable at the protein level in MDDCs, consistent with previous observations [53]. Finally, both clathrin- and caveolin-mediated endocytosis require dynamin for the scission of endosomes from the plasma membrane whereas our results indicate that dynamin is not required for VACV entry in MDDCs. Dynamin is also required for phagocytosis although its role is in the release of secretory vesicles that supply new membranes to the growing pseudopods. In macropinocytosis, the closure and scission of macropinosomes is thought to be carried out by CtBP-1/BARS and regulated by Pak1 activity [42]. There are conflicting reports about the dependence of VACV on dynamin during fluid-phase uptake in HeLa cells. One study found that MV entry was sensitive to Dynasore, DynII siRNA and the dominant negative DynI K44A mutant (but not the dominant negative DynII K44A mutant), concluding that dynamin was essential for VACV entry [45], whereas others found similarly that the DynII mutant had no effect on MV entry in the same cell type, but nor did similar concentrations of Dynasore and thus concluded that MV entry was independent of dynamin [23]. In MDDCs, we found the use of small molecule dynamin inhibitors to be more effective than transfection with siRNA or dominant negative mutants and our results with Dynasore and Bis-T-23 were consistent with the latter report, indicating a dynamin-independent uptake mechanism.

Several lines of evidence point towards a macropinocytic uptake mechanism for VACV in DCs. The dependence on ATP, actin, membrane cholesterol and PI(3)K as well as independence from dynamin are all consistent with this pathway. Furthermore, macropinocytosis has been shown to depend on a slow rise in the intracellular Ca^2+^ concentration in MDDCs [46] so the acute sensitivity of both MV and EV to treatment with the intracellular Ca^2+^ chelator, EGTA/AM, but not non-membrane permeable EGTA, is also consistent with this pathway.

We and others [48] have shown that rottlerin is a more specific inhibitor of macropinocytosis in MDDCs than either DMA or EIPA. Rottlerin was originally described as a specific inhibitor of the delta subunit of protein kinase C however it has since been shown to affect multiple kinases via a complex, indirect mechanism and is likely not to be a specific kinase inhibitor at all [54], [55]. Currently its molecular target is unknown although it does affect dynamic actin reorganisation, preventing the spreading of DCs [48]. As mentioned above, macropinocytosis depends on a slow rise in the intracellular Ca^2+^ concentration [46] and interestingly, rottlerin is known to be a potent activator of the large conductance voltage, Ca^2+^-activated K^+^ (BK) ion channel. This channel belongs to the same family as the voltage-gated K^+^ (Kv) channel which is responsible for regulating the influx of Ca^2+^ into DCs in response to maturation stimuli [56]. By activating the BK channel, rottlerin stimulates a massive outflow of current in heart and nervous tissue [57]. It is tempting to speculate that if rottlerin acts on the S4 domain that is common to both BK and Kv channels it could potentially prevent the influx of Ca^2+^ that is required for macropinocytosis to proceed, accounting for its inhibitory effect on VACV entry. Finally, both MV and EV colocalised with dextran in macropinosomes. Even though the majority of dextran is taken up via mannose receptor-mediated endocytosis, approximately 25% is macropinocytosed and cannot be blocked by mannan [38], [48]. Since mannan has no effect on the entry of MV or EV, colocalisation between the virus and dextran is likely to occur in macropinosomes.

The trafficking of macropinosomes is currently poorly understood although the destination for macropinocytosed cargo seems to depend on the nature of the cargo itself. While macropinosomes have been shown to deliver ovalbumin to distinct compartments that acquire the lysosomal protein Lamp1 and MHC class II [38], others have shown that macropinocytosed beads and dextran enter a compartment that acquires the early endosomal antigen EEA1 but they do not go on to acquire Lamp1 or MHC class II [46]. The subsequent trafficking of viruses known to enter cells via macropinocytosis has not been examined. We found that VACV does not enter the classical endolysosomal pathway in DCs, as evidenced by its lack of colocalisation with early and late endosomal and lysosomal markers. DCs maintain a neutral or only mildly acidic pH in early phagosomes and macropinosomes [58], [59] enabling the storage of antigen taken up in the periphery while the DC migrates to the lymph node. Consistent with this entry pathway are our data that suggest VACV does not access an acidic compartment or rely on low pH to enter the cytoplasm in DCs. Furthermore, the marked difference in the kinetics of entry of both VACV forms, particularly EV, between DCs and BS-C-1 cells may relate to the capacity of DCs to retain antigen during migration.

Finally, although VACV has been shown to bind to cell-surface glycosaminoglycans, this interaction has proven to be cell-type dependent and previous work suggests that the VACV receptor(s) expressed on primary haematolymphoid cells may differ from epithelial cell lines since immune sera containing antibodies which blocked VACV binding to monocytes and activated T cells did not block binding to cell lines [17]. Furthermore, the receptor for VACV on T cells can be removed by trypsin [17], as we saw with MDDCs, whereas trypsinisation of cell lines does not reduce VACV binding or infection [9]. A growing number of viruses have been shown to utilise CLRs expressed on DCs (some of which are sensitive to trypsin, such as DC-SIGN [31]) for entry, including HIV-1 [60], adenovirus serotype 5 [61], measles virus [62], hepatitis C virus [63], cytomegalovirus [64] and others. Most of the CLRs expressed on DCs contain cytoplasmic internalisation motifs [65] serving to enhance internalisation, degradation and subsequent presentation of antigen to T cells. Our finding that VACV, most notably EV with multiple glycosylated proteins in its envelope [8], does not bind to CLRs distinguishes it from these other viruses and extends its capacity for immune evasion.

In conclusion, both MV and EV forms of VACV utilise macropinocytosis for entry into MDDCs. Whilst this is the predominant entry mechanism for EV, MV was less sensitive to perturbations in cellular cholesterol levels and shut down of macropinocytosis, which suggests that it may utilise more than one dynamin-independent endocytic pathway. Our study is the first demonstration that EV can enter cells via a mechanism other than fusion with the plasma membrane. These results lay the foundation for further investigations in animal models to determine the significance of DC macropinocytosis of both MV and EV in vaccinia pathogenesis and use of vaccinia recombinants as vaccine candidates, for example, by examining the *in vivo* effects of amiloride or wortmannin-induced inhibition of macropinocytosis in mice on antigen presentation by various myeloid DC subsets [66]. Further elucidation of the fate of VACV inside DCs will contribute not only to our understanding of the biology of VACV interactions with the immune system but also the efficacy of vaccines employing VACV vectors and should assist their rational design.

## Materials and Methods

### Antibodies

The following were kind donations: MV-neutralising murine monoclonal antibody (mAb) 7D11, directed against the MV protein L1R from B. Moss (NIH, Bethesda, MD; with permission from A. Schmaljohn, USAMRIID, Frederick, MD; [67]). Murine mAb AB1.1, directed against the MV protein D8 from G. L. Smith (Imperial College, London, UK) and rat mAb 19C2, directed against the EV protein B5 from J. Krijnse-Locker (EMBL, Heidelberg, Germany [68]). Anti-GFP rabbit polyclonal Ab was from Molecular Probes (Eugene, OR), DC-SIGN mAb (AZN-D1) was from Beckman-Coulter (Fullerton, CA), flotillin-1 (Flot-1) rabbit polyclonal Ab was from Abcam (Cambridge, UK) and Flot-1 (clone 18), mannose receptor (MR; 19.2), caveolin-1 (Cav-1; 2234), EEA1 (clone 14) and Lamp2 (H4B4) mAbs, goat anti-mouse IgG (GAM)-PE and streptavidin-PE were purchased from BD Pharmingen (Franklin Lakes, NJ). Goat anti-rabbit Ig (GAR)-FITC was from Sigma-Aldrich (St. Louis, MO). GAM-546, GAR-546 and donkey anti-rat IgG (DAR)-594 were purchased from Molecular Probes. GAM-IRdye-680 was from LI-COR Biosciences (Lincoln, NB).

### Preparation of MDDCs

Monocyte-derived DCs (MDDCs) were generated by culturing human CD14^+^ cells, positively selected from PBMCs using magnetic microbeads (Miltenyi Biotec, Gladbach, Germany), in RPMI/10% FCS (RF10) with 7.5 ng/mL IL-4 and GM-CSF (ProSpec, Rehovot, Israel) for 6 days [69].

### Preparation of VACV stocks

A WR strain VACV with EGFP tagged to a core protein, vA5L-GFP-N (kindly donated by G. L. Smith, Imperial College, London [70]) was used for both MV and EV preparations. MV stocks were grown in RK13 cells for 48 h as previously described [71] and purified on a discontinuous 16–32% Optiprep (Axis-Shield, Oslo, Norway) gradient in a SW28 rotor (Beckman Coulter) at 28 000 rpm for 1 h at 4°C. Purified MV banded at the 28–32% interface which was harvested and pelleted on a 50% Optiprep cushion in a SW41 Ti rotor (Beckman Coulter) at 14 000 rpm for 45 min. The purity of the virus stock was confirmed by immunofluorescence and electron microscopy and SDS-PAGE followed by a general protein stain and western blotting for D8 and GFP proteins (Fig. S1). MV was always sonicated for 45s at 130W to disrupt any aggregates prior to infecting cells. Fresh EV stocks were grown for each experiment in BHK-21 cells as described previously [14]. Supernatants were harvested after 24 h, clarified by low speed centrifugation at 1200 rpm for 10 min to remove cellular debris and concentrated by 3 rounds of centrifugation through 100 kDa Amicon Ultra-15 filters (Millipore, Billerica, MA) at 2000 rpm for 20 min at 4°C. Any contaminating MV and damaged EV were neutralised with the 7D11 mAb at 1∶400 for 1 h at 37°C. Viruses were titered by plaque assay on BS-C-1 cells. The percentage of intact EV was calculated as the ratio of the viral titres in the presence:absence of 7D11 mAb (Fig. S2) and the presence of intact EV was confirmed by immunofluorescence microscopy as previously described (Fig. S3; [72]).

### Virus entry assay

MDDCs were spinoculated with vA5L-GFP MV (MOI 20) or EV (MOI 0.5–5), unless otherwise stated, at 650 g for 1 h at 4°C. As passive binding of VACV to MDDCs at 4°C is minimal, we used spinoculation to enhance both MV and EV binding and enable the study of entry events. Spinoculation was not found to be detrimental to cell viability, consistent with previous reports [14]. Following spinoculation, cells were retained on ice or resuspended in warm RF10 and incubated at 37°C for the indicated time to allow bound virus to enter the cells. Residual surface-bound virus was removed by treatment with 0.5% trypsin for 10 min at 37°C and cells were washed once in RF10 then twice more in ice cold PBS.

### Entry inhibition studies

Inhibitors were purchased from Sigma-Aldrich (St. Louis, MO) unless otherwise specified. Ethyleneglycol-*bis*(β-aminoethyl)-N,N,N′,N′-tetraacetoxymethyl ester (EGTA/AM) was from Calbiochem (San Diego, CA). Bis-T-23 and Dyngo7a (Dynasore) were developed in-house [73]. MDDCs were pre-treated in serum-free media in the absence or presence of inhibitor for the times and concentrations indicated. Cells were then subjected to the virus entry assay, with spinoculation and virus entry occurring in the presence of the inhibitor, and fixed in 4% paraformaldehyde (PFA). The percentage of GFP-positive cells was analysed by flow cytometry and expressed as a percentage of the no drug control (designated as 100% entry). For depletion of ATP, MDDCs were washed and resuspended in glucose-free RPMI (Invitrogen) with the addition of either 10 mM D-glucose (no drug control) or 10 mM D-deoxyglucose (Sigma-Aldrich) and 10 mM sodium azide to prevent glycolysis. Depletion of >90% of ATP was confirmed using an ATP Determination Kit (Molecular Probes) according to the manufacturer's instructions. Depletion of cellular cholesterol was confirmed using an Amplex Red assay kit (Molecular Probes) according to the manufacturer's instructions.

For inhibition of transferrin or Lucifer Yellow uptake, MDDCs pre-treated with dynamin or macropinocytosis inhibitors as described above were then incubated with warm media containing 5 µg/mL Alexafluor-647-labelled transferrin (Tf-647; Molecular Probes, Eugene, OR) or 200 µg/mL Lucifer Yellow (Sigma-Aldrich) respectively for 15 min at 37°C. Cells were placed on ice to halt endocytosis and washed three times with ice-cold PBS. Surface-bound transferrin was removed by an ice-cold acid wash (0.2 M CH_3_COOH, 0.5 M NaCl, pH 2.8) for 15 min. MDDCs were then fixed in 4% PFA and analysed by flow cytometry. The mean fluorescence intensity (MFI) of the sample minus the background MFI (unpulsed cells) was expressed as a percentage of the no drug control MFI.

### Inhibition of CLR binding

For inhibition of CLR-mediated virus binding cells were incubated in ice-cold binding buffer (RPMI 1640, 10 mM HEPES, 1% BSA; pH 7.4) with mannan, EGTA, D-mannose or neutralising anti-DC-SIGN mAb at the concentrations indicated for 40 min at 4°C. Except EGTA treated samples, cells were washed in ice-cold binding buffer to remove excess inhibitor. EGTA-treated samples were not washed in order to maintain calcium-depleted conditions. Cells were then spinoculated with vA5L-GFP MV (MOI 50) or EV (MOI 5–10) at 4°C, washed three times in ice-cold PBS and fixed with 4% PFA for analysis by flow cytometry. Alternatively, cells were resuspended in DNA lysis buffer for qPCR. Biotinylated [31], monomeric HIV-1 gp120 (SLCA-1 primary R5 strain [74]; kindly provided by Dr. J. Arthos, National Institutes of Health, Bethesda, MD) was used at 10 µg/mL in the same binding assay and detected with streptavidin-PE (0.5 µg/mL). For inhibition of HIV-1 infection, cells treated with inhibitors as above were infected with HIV-1 (BaL strain) at MOI 10 for 3 h at 37°C then washed and cultured for 72 h followed by qPCR analysis.

### RNA extraction and cDNA conversion

Cells were lysed and RNA extracted using a RNeasy Plus Mini Kit (Qiagen, Valencia, CA) according to the manufacturer's instructions. RNA was reverse transcribed into cDNA using a High Capacity cDNA Reverse Transcription Kit (Applied Biosystems, Foster City, CA).

### qPCR

Copies of the VACV DNA genome or viral transcripts were detected by primers to the virally encoded GFP (fwd: 5′-GACGTAAACGGCCACAAGTT-3′; rev: 5′-GAACTTCAGGGTCAGCTTGC-3′) or immediate early genes E3L (ORF number VACV059 in Western Reserve strain; fwd:5′-TATCCGCCTCCGTTGTCATA-3′; rev:5′-CGAAGGAGCTACTGCTGCAC-3′) and B2R (ORF number VACV184 in Western Reserve strain; fwd:5′-TGGAGCACTGCTGCCTATGT-3′; rev:5′-CTCGTACCCGATTCCGCTTA-3′) (www.poxvirus.org) using Platinum SYBR Green qPCR SuperMix-UDG (Invitrogen) in a Corbett Research Rotor-Gene (Corbett Life Sciences, Sydney, Australia) with the following cycling conditions: 50°C for 2 min, 95°C for 10 min, 40 cycles of 95°C for 15 sec and 60°C for 1 min and normalised to GAPDH as previously described [75]. HIV-1 DNA copies were quantified by detecting HIV-1 LTR-*gag* DNA using primers and a molecular beacon [76] and normalised to albumin copy number as previously described [75]. Cav-1 primers: Fwd: 5′-ACAGCCCAGGGAAACCTC-3′. Rev: 5′-GATGGGAACGGTGTAGAGATG-3′.

### Expression of Cav-1 and Flot-1 by western blot and flow cytometry

Cells were lysed at 10×10^6^/mL (10 mM HEPES, 150 mM NaCl, 1% Triton X-100, 10 mM CaCl_2_ and 100 mg/L protease inhibitor cocktail (Sigma-Aldrich)) at 4°C for 1 h. The soluble fraction was analysed for Flot-1 and insoluble fraction for Cav-1. Lysates were separated by SDS-PAGE (12% gel) and transferred to nitrocellulose membranes (Amersham Pharmacia Biotech, Arlington Heights, IL). Membranes were blocked overnight with Odyssey blocking buffer (LI-COR) and probed with Flot-1 mAb (1∶500) or Cav-1 mAb (1:5000) and GAM-IRdye-680. Membranes were imaged using the Odyssey Infra-red Imaging System (LI-COR). NIH/3T3 cells were used as a positive control. For flow cytometric analysis, MDDCs or NIH/3T3 cells were permeabilised and stained with Flot-1 pAb (5 µg/mL) followed by GAR-FITC (10 µg/mL) or Cav-1 mAb (5 µg/mL) followed by GAM-PE (10 µg/mL). The percentage of antigen-positive cells was analysed by flow cytometry.

### Confocal microscopy

#### (i) Colocalisation of VACV with cellular markers or endocytic tracers

Cells were spinoculated with vA5L-GFP MV or EV at 4°C, washed to remove unbound virus then fixed as described in each case to analyse binding or incubated in pre-warmed RF10 at 37°C to allow bound virus to enter cells. Cells were pre-incubated with 10 µg/mL MR mAb or neutralising DC-SIGN mAb prior to spinoculation or stained with EEA1 (5 µg/mL) or Lamp2 (5 µg/mL) mAbs after fixation. These markers were detected with GAM-546 (5 µg/mL). For EV-infected samples, the EEA1 and Lamp2 mAbs were directly conjugated to Alexafluor-555 using Zenon labelling technology (Molecular Probes) according to the manufacturer's instructions, prior to staining in order to avoid cross-reactivity of the secondary Ab with the murine mAb used to neutralise damaged MV in the preparation of EV stocks. Alternatively, Tf-647 (200 µg/mL) or dextran-Texas Red (0.5 mg/mL, Molecular Probes) was added to the medium during virus entry for the time specified. The cells were washed and trypsinised to remove residual surface-bound virus then washed twice more in ice-cold PBS before fixing and analysis by confocal microscopy. The extent of GFP-positive pixels that colocalised with tracer-positive pixels was quantified using Mander's co-efficient in the JACoP plug-in in Image J [77] after first masking out cells that did not take up both virus and tracer.

#### (ii) Analysis of Cav-1 and Flot-1 expression

MDDCs or NIH/3T3 cells were fixed with 2% PFA and permeabilised with 0.1% Triton X-100. Cells were then stained with Cav-1 mAb (5 µg/mL) followed by GAM-546 (5 µg/mL) or Flot-1 pAb (2.5 µg/mL) followed by GAR-546 (10 µg/mL).

Coverslips were mounted with ProLong Gold anti-fade reagent (Molecular Probes) and cell images were acquired using an upright Leica DMRE fluorescence microscope, Leica SP2 confocal system and software v2.5 (Leica Microsystems, Heidelberg GmbH, Mannheim, Germany).

### Statistical analyses

Data are presented as means ± SEM and n represents the number of experiments in independent donors. The statistical software packages SPSS for Windows, Version 14, and S-PLUS Version 6.2 were used to analyse the data for the VACV entry inhibition studies. Linear mixed effects models were used to quantify the dose response of the log transformed outcome to different drugs. Two-tailed tests with a significance level of 5% were used throughout.

## Supporting Information

Figure S1 Characterisation of purified MV stocks. (A–C) MV stocks were initially characterised using a virus with a GFP-labelled EV envelope (vB5R-GFP, kindly donated by B. Moss, NIH). (A) The viral titre of each band in an Optiprep gradient from three separate preparations of vB5R-GFP MV is shown, as determined by plaque assay. The highest titre of virus was consistently recovered from the 28–32% interface (“32%”). (B) FACS analysis of MDDCs spinoculated with virus from each band at 4°C. Virtually no GFP fluorescence was detected in cells infected with virus from the “32%” band indicating an absence of EV particles. (C) Immunofluorescence microscopy of virions from different bands from a preparation of vB5R-GFP bound to fibronectin-coated coverslips and stained with mAb AB1.1 (against MV membrane protein D8) and GAM-546. MV particles are shown in red, intact EV particles in green, and damaged EV particles in yellow (dual stained). Arrows indicate dual-stained, damaged EV in the 20% band. Arrow-heads indicate rare, green, intact EV in the 28% band. The 32% band was composed almost exclusively of red MV particles. (D) General protein stain. Purified vA5L-GFP MV (core-labelled virus) was solubilised in SDS-loading buffer and subjected to SDS-PAGE and Coomassie Blue staining. Molecular weight markers are indicated at the left. (E) vA5L-GFP MV run on SDS-PAGE as in (D) was transferred to a nitrocellulose membrane for western blotting with antibodies to the D8 (mAb AB1.1) and GFP (mAb 8362-1; Clontech) proteins. The 67 kDa band is consistent with the A5L-EGFP fusion protein. (F) Electron micrograph of purified MV on the surface of a DC.
(0.16 MB TIF)

Figure S2 Production and concentration of EV stocks. (A) Wild-type WR VACV and vA5L-GFP VACV were used to infect BS-C-1 or BHK-21 cells in a comet assay. The infected cells were overlaid with liquid medium and after 48–72 h were stained with crystal violet. vA5L-GFP produces very little EV in BS-C-1 cells compared to wild type VACV as indicated by the absence of comets, but produces EV in comparable amounts to wild type virus in BHK-21 cells, indicated by the presence of comets. (B) Electron micrograph of a triple-membraned intracellular EV inside an infected BHK-21 cell and (C) a double membraned EV released from an infected BHK-21 cell confirming the production of such virions in this cell type. (D) Supernatants from BHK-21 cells infected for 24 h were either left unconcentrated, concentrated by centrifugation in Amicon filters at 650 g for a total of 1 h or concentrated by ultracentrifugation at 19 000 g for 1 h. The resulting preparations were titred by plaque assay, with and without the addition of mAb 7D11 to neutralise MV and damaged EV. The percentage of intact EV virions was calculated as the ratio of these two titres. The data presented are the means of 7 unconcentrated, 7 Amicon and 3 ultracentrifuged preparations with standard error bars. (E) 1:4000 and 1∶400 dilutions of mAb 7D11 were tested on increasing concentrations of MV to determine whether they would be capable of neutralising an EV preparation that was entirely composed of damaged EV or MV. The percentage reduction in plaques in shown. 1∶400 dilutions of 7D11 were used in all subsequent assays.
(0.20 MB TIF)

Figure S3 Confirmation of the presence of intact EV in EV stocks. Virions from concentrated EV stocks of core-labelled vA5L-GFP VACV (green) were bound to fibronectin-covered coverslips and stained with (A) an EV-specific mAb 19C2 and donkey anti-rat-546 secondary Ab (red). EV particles were double labelled (yellow). Arrows indicate green MV particles. (B-D) Alternatively virions (green) were stained with an MV-specific mAb, AB1.1 and GAM-546 (red). (B) As a negative control, an isotype control Ab was used instead of AB1.1. (C) As a positive control, virions were permeabilised with methanol:acetone (1∶1 v/v) at −20°C for 2 min, prior to staining with AB1.1. (D) In the test sample, intact EV excluded AB1.1 staining and appear green. Arrowheads indicate double-labelled (yellow) contaminating MV or damaged EV.
(0.08 MB TIF)

Figure S4 VACV does not colocalise with Flot-1. Expression of Flot-1 in MDDCs was assayed at the protein level by (A) western blot resolving as a 48 kDa band with SDS-PAGE, (B) intracellular flow cytometry and (C) confocal microscopy. Monoclonal Flot-1 Ab was detected with GAM-IRdye-680 for western blot, polyclonal Flot-1 Ab was detected by GAR-FITC for flow cytometry and GAR-546 for confocal microscopy. NIH/3T3 cells were used as a positive control. (D) MDDCs were spinoculated with MV-GFP (MOI 10) or EV-GFP (MOI 2–5) at 4°C and the virus subsequently allowed to enter at 37°C for up to 60 min. In this case, residual surface-bound virus was not removed by trypsinisation. Cells were fixed with 2% PFA and permeabilised with 0.1% Triton X-100 then stained as for (C). Representative maximum projections of z-series taken at 15 min are shown. Scale bars represent 5 µm. Inserts are enlargements of the boxed areas in the main images.
(0.25 MB TIF)

Figure S5 The specificity of macropinocytosis inhibitors differs between cell types. (A) Rottlerin is the most specific macropinocytosis inhibitor in MDDCs based on its preferential inhibition of Lucifer Yellow uptake over transferrin-647 uptake. Cells were pretreated with rottlerin (10 µM), DMA (200 µM) or EIPA (100 µM) then incubated with Lucifer Yellow (200 µg/mL) or Tf-647 (5 µg/mL) for 15 min at 37°C. Uptake was measured by FACS and expressed as a percentage of the untreated control. (B) The effect of macropinocytosis inhibitor EIPA on the entry of MV-GFP (white bars) and EV-GFP (shaded bars) into HeLa cells was assayed by FACS, for comparison with MDDCs. HeLa cells were pretreated with EIPA at the concentrations indicated for 30 min at 37°C prior to spinoculation with MV (MOI 20) or EV (MOI 0.5-5) at 4°C and subsequent virus entry assays. Data are the means of three individual experiments with standard error bars.
(0.03 MB TIF)
