# Supplementary figures and images for: A Differential Role for Macropinocytosis in Mediating Entry of the Two Forms of Vaccinia Virus into Dendritic Cells
Source: PLoS Pathog. 2010 Apr 22;6(4):e1000866. doi: 10.1371/journal.ppat.1000866 (PMC2858709; doi:10.1371/journal.ppat.1000866)

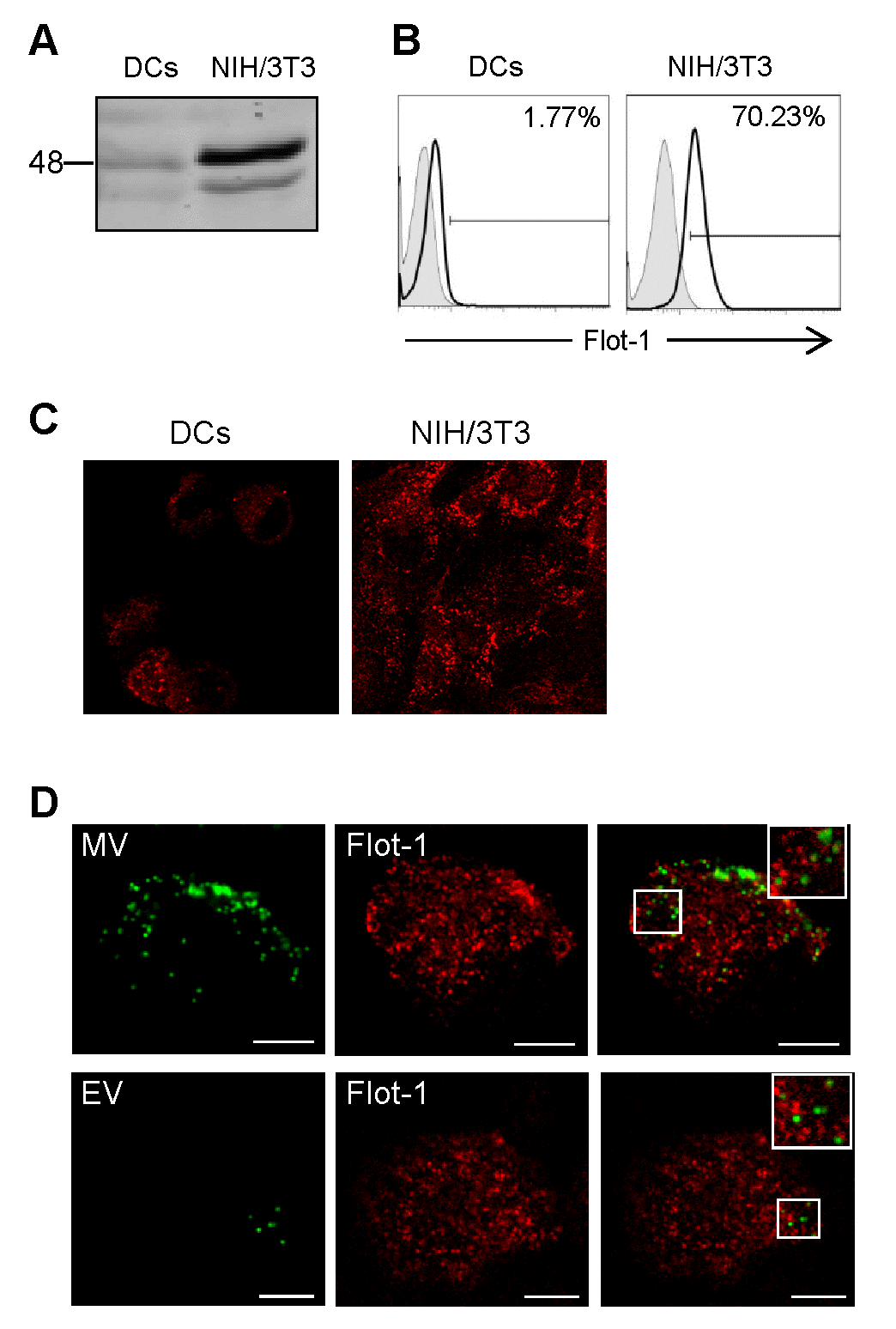

Supplement: Figure S4 — VACV does not colocalise with Flot-1. Expression of Flot-1 in MDDCs was assayed at the protein level by (A) western blot resolving as a 48 kDa band with SDS-PAGE, (B) intracellular flow cytometry and (C) confocal microscopy. Monoclonal Flot-1 Ab was detected with GAM-IRdye-680 for western blot, polyclonal Flot-1 Ab was detected by GAR-FITC for flow cytometry and GAR-546 for confocal microscopy. NIH/3T3 cells were used as a positive control. (D) MDDCs were spinoculated with MV-GFP (MOI 10) or EV-GFP (MOI 2–5) at 4°C and the virus subsequently allowed to enter at 37°C for up to 60 min. In this case, residual surface-bound virus was not removed by trypsinisation. Cells were fixed with 2% PFA and permeabilised with 0.1% Triton X-100 then stained as for (C). Representative maximum projections of z-series taken at 15 min are shown. Scale bars represent 5 µm. Inserts are enlargements of the boxed areas in the main images. (0.25 MB TIF) [file ppat.1000866.s004.tif]
